# Association between knowledge of caries preventive practices, preventive oral health habits of parents and children and caries experience in children resident in sub-urban Nigeria

**DOI:** 10.1186/1472-6831-14-156

**Published:** 2014-12-16

**Authors:** Morenike O Folayan, Kikelomo A Kolawole, Titus Oyedele, Nneka M Chukumah, Nneka Onyejaka, Hakeem Agbaje, Elizabeth O Oziegbe, Olusegun V Osho

**Affiliations:** Department of Child Dental Health, Obafemi Awolowo University, Ile-Ife, Nigeria; Oral Habit Study Group, Ile-Ife, Nigeria; Department of Child Dental Health, Obafemi Awolowo University Teaching Hospitals Complex, Ile-Ife, Nigeria

**Keywords:** Caries, Prevention, Nigeria, Fluoride, Tooth brushing, Tobacco, Sugar

## Abstract

**Background:**

The objectives of this study were to assess the association between children and parents’ knowledge of caries preventive practices, the parents’ caries preventive oral health behaviours and children’s caries preventive oral health behaviour and caries experience.

**Method:**

Three hundred and twenty four participants aged 8–12 years, 308 fathers and 318 mothers were recruited through a household survey conducted in Suburban Nigeria. A questionnaire was administered to generate information on fathers, mothers and children’s knowledge of caries prevention measures and their oral health behaviour. Clinical examination was conducted on the children to determine their dmft/DMFT. Analysis was conducted to determine the predictors of the children’s good oral health behaviour.

**Result:**

The mothers’ oral health behaviours were significant predictors of the children’s oral health behaviours. Children who had good knowledge of caries prevention measures had significant increased odds of brushing their teeth twice daily or more. The children’s caries prevalence was 13.9%, the mean dmft was 0.2 and the mean DMFT was 0.09. None of the dependent variables could predict the presence of caries in children.

**Conclusion:**

The study highlights the effect of maternal oral health behaviour on the oral health behaviour of children aged 8 years to 12 years in suburban Nigeria. A pilot study is needed to evaluate how enhanced maternal preventive oral health practices can improve the oral health preventive practices of children.

## Background

Individual’s oral health-related behaviours and their outcomes are influenced by individual, family and community level factors [1]. These same factors, to a great extent, determines the individuals’ lifestyle [2–6]. An important family level factor is the socio-economic status. Important community level factors are those associated with ease of access to health care [7]. At an individual level, age, racial and ethnic group, education, and gender play important roles [8–13]. These factors continuously interact to shape the human behaviour including those that affect the adoption of new oral health habits learnt through the traditional knowledge-attitude-behaviour chain [14–16].

Children’s health-related attitude and behaviours are taught and adopted at home through a process called primary socialization. Later, these attitude and behaviours are shaped and formalized through the community network formed with friends, peers and teachers and significant others the children interact with through a process called secondary socialization [16]. It is therefore important to understand how significant the primary and secondary socialization processes are in the development of oral health behaviour of children in various cultures and communities. This would help inform the design of cost effective oral health education programmes for children. As a first step however, it is important to understand the prevention oral health care practices of children in various communities.

Very little is known about the prevention oral health care practices of children in Nigeria especially the use of a combination of fluoridated toothpaste once a day or more and restricted intake of refined carbohydrate for caries risk reduction. Multiple studies had shown that most children in Nigeria brush their teeth once a day [17]. A prior study had shown that the use of fluoride containing toothpaste is widespread [18] and less than a third of school aged children consumed sugar less than once a day [18]. Only 7.8% of school age children use a combination of caries risk prevention tools (consumption of refined carbohydrate once a day, continuous use of fluoridated toothpaste once a day or more, and restricted intake of refined carbohydrate) [18]. However, the ability to generalize the findings of these studies conducted among in-school children is limited since as high as 40% of primary school children and 60% of secondary school children are out-of school [19]. Studying the use of a combination of caries prevention approaches is important as a combination of approaches enhances behaviour dependent interventions [20]. No prior studies had tried to assess association between parental oral health practices on the oral health preventive practices of children in the study environment despite the known role of the family in establishing early oral health behaviours [16].

Our study therefore tried to address some of these gaps in knowledge. We tried to determine the proportion of children who use a combination of caries prevention approaches in Ile-Ife Nigeria, a sub-urban community in Nigeria, using a more robust method than the previous study. In addition, we also determined factors associated with preventive oral health care practices of children in the study location. Specifically, the study examined the association between the child and parents’ caries preventive oral health practices, and the association between children’s caries preventive oral health practices and the presence of caries. It also assessed the association between children and their parents’ knowledge of caries preventive practices and the children’s caries preventive practices.

## Methods

### Study design

This study was part of a larger study conducted to explore the relationship between oral habits and caries in children age 6 months to 12 years. It was a cross sectional study which recruited study participants through a household survey. A household survey was conducted in view of the fact that not all children in Nigeria are in school. Nigeria has the highest number of children out of school in the world [21]. Data was collected in August 2013 when the children were on holidays. Data collection also took place over the weekends when parents were expected to also be at home and could participate in the study.

### Study setting

The study was conducted in Ife Central Local Government Area (LGA) of Osun State, a semi-urban area. Ife Central was chosen as the study location due to the proximity of the area to the dental school where the study team was domiciled. The 2006 National Census figures put the population of the Local Government Area at 167,204. The estimate of the child population for the Local Government is 14,000: about 10% of the total population.

### Study population

The study population included children who were 8 years to 12 years old, whose parents gave consent for study participation, and who were at home at the time data were collected. Any child who had any form of cognitive impairment or who did not give assent to participate in the study was excluded from the study.

### Sample size

The sample size for the main study was determined by using the menu of the Computer Programme for Epidemiologists (PEPI) version 3.01. The menu uses the sample size formula for estimation of single proportion as described by Armitage and Berry and cited in Gahlinger and Abramson [22]. The minimum sample size for the study was 202 where the prevalence for caries was 13.9% [23], α was 0.05 and Z was 1.96 at the 2-tailed level. The maximum acceptable difference from the true proportion was 5.0%.

### Sampling technique

The sampling procedure was a (three-level) multi-stage cluster sampling aimed at selecting eligible persons with known probability. Stage 1 involved the random selection of eight out of the 25 enumeration areas within Ife Central LGA. The 25 enumeration sites were designated by the National Populations Commission during the 2006 National Census exercise. The sites had been used for the 2009 National Demographic Health Survey [24], the 2010 National Ante Natal Clinic Sero-sentinel Survey [25] and the 2012 National Adolescent Reproductive Health Survey [26]. Enumeration sites were selected for conduct of the study because it was assumed that participants in these geographical sites may be familiar with the conduct of such surveys and thus may likely be more open to holding personal discussions with the field workers. Eight out of the 25 enumeration sites were randomly selected by balloting for this study. The number of enumeration sites for the study was limited to eight since the study had four study teams. It was proposed to have each team visit two enumeration sites.

Stage 2 involved the selection of eligible households within the enumeration sites for the survey. At each of the enumeration sites, every third household on each street was considered eligible for study participant recruitment.

Stage 3 involved the selection of actual respondents for interview and testing. Only one eligible child in each household participated in the study. Alternative sexes and age range identified for study recruitment were selected to participate in each consecutive household. Study participant recruitment continued in the enumeration site until the study sample per each data collector was reached.

### Data collection tool

Data was collected through personal interview using a structured questionnaire. Experienced field workers who had been engaged in past national surveys of this nature were employed for the study. The field workers were trained centrally on the study protocol, the use of the data collection tools, sample selection, consenting process, and the administrative process for the field work. The field workers collected information from the respondents, edited the questionnaire in the field and submitted the completed questionnaires to the survey supervisor daily. The supervisor reviewed all filled questionnaires and raised queries where gaps were identified in the filled questionnaire, or the consenting process. The queries were addressed latest by the next day by the field worker where this was feasible.

The questionnaire asked details on the child’s age and gender. Information was also collected on the preventive oral health behaviour of the child, the mother and the father respectively, respondents’ knowledge of preventive caries measures and their preventive caries behaviour. Responses were collected independently from the child, the mother and the father.

### Knowledge of caries preventive measures

The authors used the same methodology adopted by Folayan et al. [27] to analyse respondents’ knowledge of caries preventive measures. Respondents were asked to react to eight statements regarding various aspects of caries diagnosis and prevention on a five-point Likert scale ranging from 'strongly agree’ to 'agree’, 'disagree’, 'strongly disagree’ and 'do not know’. The statements were: (i) Fluoridation of drinking water is an effective, safe, and efficient way to prevent dental caries (ii) Use of fluoride containing toothpaste is an effective safe, and efficient way to prevent holes from forming on the teeth (iii) Frequency of sugar consumption has a greater role in producing caries than the total amount of sugar (iv) Sealant is effective in the prevention of pit and fissure caries in newly erupted molars (v) Rinsing teeth with a little amount of water after tooth-brushing increases the effect of fluoride (vi) Using fluoride toothpaste is more important than the brushing *per se* for preventing caries (vii) Brushing twice daily with fluoride containing toothpaste is effective for preventing holes from developing in the teeth (viii) It is important to visit the dental clinic regularly as a measure for preventing holes from forming in the teeth. For each of the eight statements, respondents who indicated 'strongly agree’ and 'agree’ as options were graded as having responded correctly to the statement. The responses were then scored from one to five with 'strongly agree’ scoring 5 and 'do not know’ scoring 1. Where there were no responses, a score of 1 was allocated.

Each respondent could therefore obtain a total minimum score of 8 and a total maximum score of 40. The mean scores for each respondent was calculated. This was used as the final knowledge score for each respondent. In order to dichotomise the variable, the median of the final scores served as cut-off point, with respondents scoring below the median categorised as having poor knowledge and all others scoring the median score and above comprising those with good knowledge. The median score for this sample was 21.

### Oral health behaviour

The authors used the same methodology adopted by Folayan et al. [17] to analyse the oral health behaviour of respondents (parents and child). The respondents were requested to report the frequency with which they brush their teeth, use fluoridated toothpaste, floss and eat sugary snacks between main meals. These questions were used to determine self-care levels. These questions had four to seven alternatives. In order to define acceptable levels of each of the components, the following cut-off points were used: brushing more than once a day, using fluoridated toothpaste always or almost always, flossing at least once a day, and eating sugary snacks between main meals less frequently than once a day. The respondents were also asked to indicate the time of the last check-up (with the alternatives: *within the last 6 months, more than 6 months to one year ago, more than 1 to 2 years ago, more than 2 to 5 years ago, more than 5 years, never, do not remember*). Attending a dental check-up within the last year was defined as preventive care use. The questionnaire also requested information on the respondents’ cigarette smoking habits. The question had six alternatives.

Respondents who chose the options '*irregularly or never*, *Once a week*, *a few* (*2*–*3*) *times a week*; *once a day*’ when asked the question on tooth-brushing, were classified as not having undertaken preventive dental care. Those who chose the options '*quiet often*, *seldom*, *not at all*’ when asked the question on use of fluoridated toothpaste were classified as not having undertaken preventive dental care. Those who chose the options '*About 3 times a day or more*, *about twice a day*, *about once a day*’, when asked the question on consumption of sugary snacks between meals, were classified as not having undertaken preventive dental care. Those who chose the options '*Not at all*, *occasionally*, *a few* (*2*–*3*) *times a week*’ when asked the question on the use of dental floss were classified as not having undertaken preventive dental care. Those who chose the options '*more than 1 to 2 years ago*, *more than 2 to 5 years ago*, *more than 5 years*, *never*, *do not remember*’ when asked the question on dental service utilization were classified as not having undertaken preventive dental care. Those who reported no current smoking habits were considered as non-smokers. All those who chose options '*Yes*, *once a month or less*, *Yes*, *a few times* (*2*–*3*) *a month* when asked questions on smoking habits were categorised as smokers*.*

Recommended oral self-care was defined as a composite score derived from indications of brushing teeth at more than once a day, use of fluoridated toothpaste, and consumption of sugary snacks between main meals less frequently than once a day [17]. Each respondent had to have met the three criteria to be categorised as practicing recommended oral self-care.

### Caries assessment

The numbers of decayed, filled and missing teeth (dmft and DMFT) were noted for children with caries. The dmft/DMFT was determined based on the WHO Oral Health Survey methods [28]. The examination for dental caries was conducted with a plain mouth mirror using a light source from a torch with the child seated on a chair. The teeth were not dried before examination but gross debris was cleared with gauze where necessary. The examination of the teeth was done in an orderly manner from one tooth or tooth space to the adjacent tooth or tooth space. Examination for dental caries included all surfaces. To arrive at a dmft/DMFT score for an individual child, three values were determined: the number of teeth with carious lesions, the number of extracted teeth due to caries, and the number of teeth with fillings or crowns [29]. Parents of children were asked to explain the loss of any teeth that was not found during the oral examination. Only tooth extracted due to caries were recorded as missing tooth. The number of teeth are summed together to give the DMFT score for the permanent dentition and the dmft score for the primary dentition. For logistic regression purposes, caries was classified as present or not present.

### Data analysis

Chi-square test was used to test associations between the recommended oral self-care knowledge and behaviour of parents and that of the child. Association between the use of recommended oral self-care and severity of caries was also tested. Binary logistic regression models were fitted to the data to calculate odds ratios (OR) and confidence intervals (95% CI) for each of the four oral self-care measures (tooth brushing more than once a day, intake of sugary snacks less than once a day, regular use of fluoride toothpaste, and use of dental floss every day or more). The dependent variable was the use of recommended oral self-care behaviour of the child. The independent variables for the model were gender, age and socioeconomic status of the child; knowledge of caries prevention scores of the child, mother and father respectively; and the recommended oral self-care behaviour of the mother and father respectively. Age was dichotomised using the median age and scores as the point of dichotomisation. For this study, the median age was 10 years. Association between the dependent variables and the child’s recommended oral self-care practice were also assessed. STATA version 10 was used for data processing and statistical analysis.

### Ethical consideration

Before commencing the study, ethical approval for the study was obtained from the Health Research Ethics Committee of the Obafemi Awolowo University Teaching Hospitals’ Complex Ile-Ife. Permission for study conduct was also obtained from the Ife Central Local Government. Efforts were made to seek appropriate community entry with advocacy visits paid to community heads and leaders. Approval for conduct of the study in their community was obtained prior to commencement of the study. Informed consent was obtained from the parents of each study participant prior to enrollment. All children were also asked to provide assent. No monetary compensation was paid to study participants. All study participants however received a gift of a small tube of toothpaste, an exercise book or a pencil at the end of study participation. All children recruited into the study received these gifts irrespective of whether they assented to participate or not. The value of each gift for the child was less than N50.00 ($0.33). All households were left with an education leaflet about caries: its aetiology and prevention.

## Results

### Profile of respondents

Three hundred and twenty four participants aged 8 years – 12 years were recruited for the study. This included 150 males and 174 females. The mean age ± (SD) of the child respondents was 9.7 ± (1.33) years. Also 308 fathers and 318 mothers of the child respondents participated in the study.

### Knowledge of caries preventive measures

180 (58.4%) fathers, 196 (61.6%) mothers and 69 (21.3%) children had good knowledge of caries preventive measures. See Table 1. There was a significant association between father, mother and child’s knowledge of caries preventive measures: A larger number of children who had good knowledge of caries preventive measures (68.1%) had fathers who had good knowledge of caries preventive measures (p = 0.02). Also, a larger number of children who had good knowledge of caries preventive measures (73.9%) had mothers who had good knowledge of caries preventive measures (p = 0.01). Although the odds of the mother’s knowledge of caries preventive measures being predictive of the child’s knowledge of caries preventive measures (74%) was higher than that of the father’s knowledge of caries preventive measures being predictive of the child’s knowledge of caries preventive measures (47%), neither of the predictive values were statistically significant. See Table 2.

**Table 1:** **Profile of the fathers, mothers and children recruited into the study**

Variables	Variables		
	Father	Mother	Children
	N = 308	N = 318	N = 324
Good knowledge of caries prevention practices	180 (58.4%)	190 (59.7%)	69 (21.3%)
Brushes teeth more than once a day	48 (15.6%)	51 (16.0%)	29 (9.0%)
Use fluoridated toothpaste always or almost always	286 (92.9%)	260 (81.8%)	270 (83.3%)
Eat sugar-containing snacks less than once a day	233 (75.6%)	234 (73.6%)	93 (30.2%)
Practice recommended oral self-care	38 (12.3%)	26 (8.2%)	6 (1.9%)
Floss at least once a day	12 (3.9%)	21 (6.6%)	7(2.2%)
Had dental check-up within last 12 months	7 (2.3%)	11 (3.5%)	8 (2.5%)
No present smoking habit	302 (98.1%)	313 (98.4%)	311 (96.0%)

**Table 2:** **Predictors of good knowledge of caries prevention practices and preventive oral health practices by children in Ile-Ife, Nigeria**

Variables	Variables		
	Odds ratio	p-Value	Confidence interval
Knowledge of caries prevention practices			
Father’s knowledge of caries prevention practices	1.47	0.25	0.76 – 2.85
Mothers’ knowledge of caries prevention practices	1.74	0.12	0.87 – 3.47
Brushes more than once a day			
Father brushes more than once a day	2.29	0.18	0.69 – 7.57
Mother brushes more than once a day	21.25	*0.000	8.56 – 52.74
Use fluoridated toothpaste always or almost always			
Father use fluoridated toothpaste always or almost always	1.14	0.83	0.35 – 7.57
Mother use fluoridated toothpaste always or almost always	39.45	*0.000	8.56 – 52.74
Eat sugar-containing snacks less than once a day			
Father eats sugar containing snacks less than once a day	0.80	0.40	0.47 – 1.35
Mother eats sugar containing snacks less than once a day	3.07	*0.000	1.64 – 5.78
Practice of recommended oral self-care (ROSC)			
Father practices ROSC	2.67	0.45	0.21 – 33.;21
Mother practices ROSC	77.15	*0.000	0.31 – 716.31
Floss at least once a day			
Father floss at least once a day	-	-	-
Mother floss at least once a day	5.30	*0.05	1.00 – 28.12
Dental check-up within last 12 months			
Father does dental checkup within last 1 year	1.01	0.09	1.00 – 1.03
Mother does dental checkup within last 1 year	-	-	-
No present smoking habit			
Father has no present smoking habit	1.34	0.79	0.16 – 10.96
Mother has no present smoking habit	6.24	*0.03	1.19 – 32.74

*statistically significantly different.

### Oral health behaviour

Tables 2 and 3 provides an overview of the oral health behavior of respondents.

**Table 3:** **Oral health behaviour of fathers, mothers and children**

Variables	Variables			p value
	Father N = 308	Mother N = 318	Child N = 324	
Brushes more than once a day				
Yes	48(15.6%)	51 (16.0%)	29 (9.0%)	0.01
No	260 (84.4%)	267 (84.0)	296 (91.0%)	
Use fluoridated toothpaste always or almost always				
Yes	286 (92.9%)	260 (81.8%)	270 (83.3%)	0.000
No	22 (7.1%)	58 (18.2%)	54 (16.7%)	
Eat sugar-containing snacks less than once a day				
Yes	233 (75.6%)	234 (73.6%)	98 (30.2%)	0.000
No	75 (24.4%)	84 (26.4%)	226 (69.8%)	
Practice of recommended oral self-care				
Yes	38 (12.3%)	26 (8.2%)	6 (1.9%)	0.000
No	270 (87.7%)	292 (91.8%)	318 (98.1%)	
Floss at least once a day				
Yes	12 (3.9%)	21 (6.6%)	7 (2.2%)	0.02
No	296 (96.1%)	297 (93.4%)	317 (97.8%)	
Dental check-up within last 1 year				
Yes	7 (2.3%)	11 (3.5%)	8 (2.5%)	0.62
No	301 (97.7%)	307 (96.5%)	316 (97.5%)	
No present smoking habit				
Yes	302 (98.1%)	313 (98.4%)	311 (96.0%)	0.11
No	6 (1.9%)	5 (1.6%)	13 (4.0%)	

Most fathers (82.5%), mothers (82.4%) and children (88.3%), reported once a day tooth brushing respectively. Significantly less children reported brushing their teeth two times a day or more when compared with their fathers (p = 0.01) and mothers (p = 0.006). Tooth brushing two times a day or more by the mother was a significant predictor of tooth brushing two times a day or more by the child: the odds of the child brushing the tooth two times a day or more increased by over 21 folds (8.6-52.7; p < 0.001) when the mother brushed twice a day or more. Although twice daily brushing or more by the father increased the odds of the child using fluoridated toothpaste by over two folds, this effect was not significant (p = 0.18).

Most fathers (92.9%), mothers (81.8%) and children (83.3%), use fluoride containing toothpastes. Significantly more fathers reported the use of fluoridated toothpastes when compared to the children (p < 0.001). There was no significant difference in the use of fluoridated toothpastes between mothers and children (p = 0.60). The use of fluoridated toothpaste by the mother was a significant predictor of use of fluoridated toothpaste by the child: the odds of the child using fluoridated toothpastes increased by over 39 folds (18.2-89.7; p < 0.001) when the mother uses fluoridated toothpaste. Although the use of fluoridated toothpaste by the father increased the odds of the child using fluoridated toothpaste by over one fold, this effect was not significant (p = 0.83).

Eating of sugar containing snacks less than once a day was least prevalent in children (30.2%) when compared to their fathers (75.6%) and mothers (73.6%). Consumption of sugar containing snacks less than once a day by mothers was predictive of consumption of sugar containing snacks less than once a day by the child: the odds of the child consuming sugar containing snacks less than once a day when the mother also consumes sugar containing snacks less than once a day was over three folds (p < 0.001).

The use of recommended caries risk reduction self-care measure was low in this study population. Only 12.3%, 8.2% and 1.9% of fathers, mothers and children in this study population used the recommended caries risk reduction self-care measure respectively. The use of recommended caries risk reduction self-care measure by the mother was a significant predictor of use of recommended caries risk reduction self-care measure by the child: the odds for use of recommended caries risk reduction self-care measure by the child when the mother uses recommended caries risk reduction self-care measure increased by over 77 folds (p < 0.001). Although the use of recommended caries risk reduction self-care measure by fathers increased the odds of use of recommended caries risk reduction self-care measure by the child by over two folds this effect was not statistically significant (p = 0.45).

The use of dental floss was extremely low in the study population. The use of dental floss by children was also much lower than its use by fathers (3.9% vs 2.2%; p = 0.20) and mothers (6.6% vs 2.2%; p = 0.006). The use of dental floss by the mothers was a significant predictor of the use of dental floss by the child: the odds of the child using a dental floss when the mother uses a dental floss increases by over five folds (p = 0.05).

Dental service utilization was also extremely poor in the study population. Only 2.3% of fathers, 3.5% of mothers and 2.5% of the children had visited the dental clinic in the last 12 months of the study. Neither the mothers’ nor the fathers’ attendance at a dental clinic in the last 12 months was a predictor of the child’s visit to the dental clinic during the same period.

Smoking was low in this study population. Only 1.9%, 1.6% and 4.0% of fathers, mothers and children in this study population smoke tobacco respectively. Having a mother not smoke tobacco significantly increased the odds of the child also not smoking tobacco by over six folds (p = 0.03). The non-smoking of tobacco by the father was not a significant predictor of the child not smoking tobacco (p = 0.79) though it increased the odds of non-smoking of tobacco by the child by more than one fold.

### Knowledge of caries preventive measures and oral health behaviour

Table 4 gives a summary of the effect of fathers, mothers and children’s knowledge of caries prevention on oral health behaviour. The fathers’, mothers’ and children’s knowledge of caries prevention were not predictors of the child’s daily use of fluoridated toothpaste, consumption of sugar containing snacks less than once a day, daily use of dental floss, utilization of the dental clinic at least once every 12 month, and the smoking of tobacco. While the father and mother’s knowledge were not significant predictors of the use of toothbrush twice daily or more, that of the children did significantly predict its use: children who had good knowledge of caries prevention measures had almost three folds increased odds of brushing their teeth twice daily or more (p = 0.009).

**Table 4:** **Knowledge of caries prevention practices as predictor of use of preventive oral health practices by children in Ile-Ife, Nigeria**

Variables	Variables		
	Odds ratio	p-Value	Confidence interval
Brushes more than once a day			
Father’s knowledge of caries prevention practices	0.93	0.88	0.36 – 2.39
Mothers’ knowledge of caries prevention practices	1.13	0.81	0.42 – 2.99
Children’s knowledge of caries prevention practices	2.92	*0.009	1.30 – 6.55
Use fluoridated toothpaste always or almost always			
Father’s knowledge of caries prevention practices	1.52	0.23	0.76 – 3.07
Mothers’ knowledge of caries prevention practices	0.96	0.92	0.48 – 1.95
Children’s knowledge of caries prevention practices	1.58	0.27	0.70 – 3.56
Eat sugar-containing snacks less than once a day			
Father’s knowledge of caries prevention practices	1.03	0.90	0.59 – 1.84
Mothers’ knowledge of caries prevention practices	0.93	0.81	0.52 – 1.67
Children’s knowledge of caries prevention practices	0.78	0.41	0.42 – 1.42
**Practice of recommended oral self-care			
Father’s knowledge of caries prevention practices	0.94	0.95	0.14 – 6.40
Mothers’ knowledge of caries prevention practices	3.60	0.30	0.33 – 39.85
Children’s knowledge of caries prevention practices	0.62	0.67	0.07 – 5.55
Floss at least once a day			
Father’s knowledge of caries prevention practices	0.30	0.21	0.05 - 1.99
Mothers’ knowledge of caries prevention practices	0.67	0.64	0.12 – 3.63
Children’s knowledge of caries prevention practices	1.61	0.58	0.31 – 8.42
Dental check-up within last 12 months			
Father’s knowledge of caries prevention practices	0.43	0.29	0.09 – 2.02
Mothers’ knowledge of caries prevention practices	2.04	0.39	0.41 – 10.15
Children’s knowledge of caries prevention practices	1.61	0.92	0.22 – 5.47
No present smoking habit			
Father’s knowledge of caries prevention practices	0.34	0.14	0.08 – 1.43
Mothers’ knowledge of caries prevention practices	2.74	0.14	0.71 – 10.56
Children’s knowledge of caries prevention practices	0.40	0.13	0.12 – 1.30

*statistically significantly different.

### Knowledge of caries preventive measures and oral health behaviour and caries risk

Only 45 (13.9%) children had caries in this study population. The dmft ranged between 1 and 4 while the DMFT also ranged between 1 and 4. The mean dmft was 0.2 and the mean DMFT was 0.09. All the six children who used the recommended caries risk reduction self-care measure did not have caries. This finding was however not statistically significant when compared with children who did not use the recommended caries risk reduction self-care measure (p = 0.32). Table 5 shows the possible effect of fathers, mothers and children’s knowledge of caries prevention, and the use of caries preventive measures on presence of caries in the mouth of the children. None of these factors were predictive of absence of caries in this study population.

**Table 5:** **Predictor of presence of caries in the oral cavity of in Ile-Ife, Nigeria**

Variables	Variables		
	Odds ratio	p-Value	Confidence interval
Father’s knowledge of caries prevention practices	1.06	0.89	0.48 – 2.32
Mothers’ knowledge of caries prevention practices	1.17	0.70	0.52 – 2.60
Children’s knowledge of caries prevention practices	0.99	0.97	0.44 – 2.21
Children brush their teeth twice a day or more	2.67	0.07	0.93 – 7.63
Children use of fluoridated toothpaste always	0.76	0.52	0.32 – 1.77
Children eat sugar-containing snacks less than once a day	1.34	0.43	0.64 – 2.30
Children floss at least once a day	2.60	0.28	0.46 – 14.78
Children had dental check-up within last 12 months	1.08	0.56	0.83 – 1.40

## Discussion

The main summary of the findings of this study is the significant effect mothers’ oral health behaviour had on the oral health behaviour of children in the study community. The daily use of fluoride containing toothpastes, tooth brushing twice daily or more, consumption of sugar containing snacks less than once a day, daily use of dental floss, non-smoking of tobacco and use of recommended caries risk reduction self-care measures by the mother significantly increased the odds of the child also doing the same things. The only preventive oral habit that the mother’s habit had no significant effect on was the utilization of dental services for oral health care in the last 12 months of the survey. While the fathers and the children’s good knowledge of caries preventive measures had no impact on the child’s preventive oral health behaviour, the child’s knowledge significantly increased the odds of their brushing twice daily or more. Neither the fathers’, mothers’ and children’s knowledge of caries preventive measures nor the use of preventive self-care measures by children in this study population were predictors of the presence of caries in the study population.

The findings of this study once again highlight the significant role of mothers in promoting adoption of caries risk preventive measures by children. Therefore oral health education programmes designed and conducted for children in this study environment needs to actively integrate mothers into the oral health care education programme. Folayan et al. [30] had proposed in their analysis of the national response to the caries epidemic in children in Nigeria that the oral health care programme for children should seek to integrate activities into the current national maternal and child response programmes. The finding of this study provides evidence to substantiate their proposal.

Prior studies had also highlighted the significant role of mothers in reducing the caries risk of children. The outcome of Shearer et al’s [31] 27-year-long study suggests that mothers with poor oral health are likely to have children who also have poor oral health when they become adults. Adeniyi et al. [32] had also highlighted that maternal factors do affect the oral health status of their children. This study only further reinforces the findings of these studies.

An important finding in this study is that as high as 4.0% of 8-12 year old children in the study community smoke tobacco; a prevalence higher than what is reported for the parents. This finding makes it important for dentists to enquire about smoking habits from teenagers during their dental visits. Institution of tobacco cessation programme early in the teenage years increases the likelihood of tobacco use cessation [33]. This is important for the prevention of oral carcinoma.

One of the strengths of this study is the methodology. A household survey was conducted thus increasing the chances of including both in- and out-of-school children of all age groups and socioeconomic class. The findings of this study can therefore be generalized to the study community. The study also provides evidence to support an earlier postulation by Folayan et al. [30] on the programmes needed to address the caries epidemic in children in Nigeria thus providing evidence for policy review and oral health programme implementation in Nigeria.

A limitation of the study however, is the wide confidence intervals observed for some significant results reported in this study. The wide confidence intervals must have resulted from the small sample for the subgroup analysis. A study using a larger sample size would be required to substantiate those findings.

In conclusion, the study highlights that maternal oral health behaviour is the most significant factor that had effect on the oral health behaviour of children aged 8 years to 12 years in Ife Central Local Government. This study once again highlights the important role of mothers in helping children develop good oral health practices in the study environment. The oral health knowledge of the children also helps increase twice daily tooth brushing by children in the study location. It is important to design a pilot study to learn how the study findings can be translated into oral health programmes in the study community.
