# Erratum: Association between knowledge of caries preventive practices, preventive oral health habits of parents and children and caries experience in children resident in sub-urban Nigeria

**DOI:** 10.1186/s12903-015-0044-4

**Published:** 2015-05-20

**Authors:** Morenike O Folayan, Kikelomo A Kolawole, Titus Oyedele, Nneka M Chukwumah, Nneka Onyejaka, Hakeem Agbaje, Elizabeth O Oziegbe, Olusegun V Oshomoji

**Affiliations:** Department of Child Dental Health, Obafemi Awolowo University, Ile-Ife, Nigeria; Oral Habit Study Group, Ile-Ife, Nigeria; Department of Child Dental Health, Obafemi Awolowo University Teaching Hospitals Complex, Ile-Ife, Nigeria

## Erratum

After publication of this research article [1], we noticed a mistake in the names of two of the authors. Olusegun V Osho name should be Olusegun V Oshomoji, and Nneka M Chukumah name should be Nneka M. Chukwumah. This has been now corrected. We apologise for any inconvenience.
